# Engineering polyphenol-based carriers for nucleic acid delivery

**DOI:** 10.7150/thno.81604

**Published:** 2023-05-21

**Authors:** Mingju Shui, Zhejie Chen, Yi Chen, Qin Yuan, Hongyi Li, Chi Teng Vong, Mohamed A. Farag, Shengpeng Wang

**Affiliations:** 1 State Key Laboratory of Quality Research in Chinese Medicine, Institute of Chinese Medical Sciences, University of Macau, Macao SAR 999078, China.; 2 Macao Centre for Research and Development in Chinese Medicine, University of Macau, Macao SAR 999078, China.; 3 Institute of Molecular Medicine (IMM), Shanghai Key Laboratory for Nucleic Acid Chemistry and Nanomedicine, Renji Hospital, School of Medicine, Shanghai Jiao Tong University, Shanghai 200127, China.; 4 Pharmacognosy Department, College of Pharmacy, Cairo University, Kasr el Aini St., Cairo 11562, Egypt.

**Keywords:** gene therapy, polyphenols, nucleic acid, RNA, EGCG

## Abstract

Gene therapy, an effective medical intervention strategy, is increasingly employed in basic research and clinical practice for promising and unique therapeutic effects for diseases treatment, such as cardiovascular disorders, cancer, neurological pathologies, infectious diseases, and wound healing. However, naked DNA/RNA is readily hydrolyzed by nucleic acid degrading enzymes in the extracellular environment and degraded by lysosomes during intracellular physiological conditions, thus gene transfer must cross complex cellular and tissue barriers to deliver genetic materials into targeted cells and drive efficient activation or inhibition of the proteins. At present, the lack of safe, highly efficient, and non-immunogenic drug carriers is the main drawback of gene therapy. Considering the dense hydroxyl groups on the benzene rings in natural polyphenols that exert a strong affinity to various nucleic acids via hydrogen bonding and hydrophobic interactions, polyphenol-based carriers are promising anchors for gene delivery in which polyphenols serve as the primary building blocks. In this review, the recent progress in polyphenol-assisted gene delivery was summarized, which provided an easily accessible reference for the design of future polyphenol-based gene delivery vectors. Nucleic acids discussed in this review include DNA, short interfering RNAs (siRNA), microRNA (miRNA), double-strand RNA (dsRNA), and messenger RNA (mRNA).

## 1. Introduction

Gene therapy is an intracellular approach of introducing exogenous therapeutic genes (transgenes) into specific host cells to elicit therapeutic effects for the treatment of genetic-based diseases via correcting the mutated/altered genes or providing the cells with a new function, which represents a promising and specific treatment for a series of diseases, such as cardiovascular, cancer, neurological, infectious diseases, and wound healing 1-5. Owing to the broad potential and guaranteed physiological activities, gene therapy has been increasingly applied for more than 3 decades, and the first clinical trial of adenosine deaminase (ADA) gene therapy for the treatment of severe combined immunodeficiency (SCID) was in 1990 6. Generally, gene therapy can be divided into two major types, somatic and germline therapies, which brings new possibility and treatment options to multiple medicinal fields 1,7. However, as naked DNA/RNA molecules can be degraded easily by intracellular lysosomes or extracellular nucleic acid degrading enzymes, gene transfer must cross the multiple cellular and tissue barriers to deliver genetic materials into the pathological sites and trigger efficient expression of the therapeutically bioactive substances or obvious down-regulation of proteins 8,9. To achieve effective delivery of gene based-drugs, innovative preparations that are characterized by excellent cell targeting specificity, gene expression regulation, gene transfer efficiency, and vector safety, are warranted 10.

Currently, gene delivery systems can be categorized into viral and non-viral systems. Due to the extreme efficiency at transferring genes, viral vectors, such as retrovirus, adeno-associated virus, adenovirus, pox virus, lentivirus, herpes simplex virus, and human foamy virus (HFV), are regarded as one of the significant constituent bases of gene therapy systems 11. Nevertheless, safety concerns about severe toxicity and off-target immunogenicity have severely limited the clinical translation of viral gene delivery 12. Although recombinant viruses are non-pathogenic powerful vectors, it is still possible to revert the virus to wild-type virus or co-purify it with replicating virus 13. In addition, viral vectors can elicit immunogenic responses and induce the activation of inflammatory system, toxin production, and insertional mutations, which can lead to cancer 14,15. Other bottlenecks of the low targeting specificity and high manufacturing costs also further hinder the application of viral vector-based gene therapy 13,16. All these safety issues and existing challenges motivate the exploration of safer, less immunogenic and pathogenic, highly efficient, and stable gene delivery vectors. Non-viral vectors, also called synthetic vectors, are widely developed to deliver genetic payloads 13. Non-viral vectors are safer and more flexible than viral vectors, and provide greater structural and chemical versatility for exploiting physicochemical properties 10,17. Although the efficiency of non-viral vectors in gene delivery is less than viral vectors, the lower toxicity, higher vector stability, larger gene capacity, lower cost, and less immunogenic response also make them to be more reliable for gene therapy 9,12. The synthetic methods of non-viral vectors include physical and chemical methods, such as electroporation, ultrasound, magnetofection, gene gun, cationic polymers and liposomes 18,19. In clinical trials, two non-viral gene delivery methods are widely utilized, including direct injection of the naked gene drugs (plasmids containing the transgene) into the tissues and the preparation of lipofection to pack nucleic acids 20. With the advancement of drug delivery system and nanotechnology, a variety of non-viral nanocarriers were designed for gene delivery, such as cationic lipids, polymers, graphene, dendrimers and other inorganic nanoparticles, which rely on three main packaging strategies, including electrostatic interaction, encapsulation and adsorption 10,21,22. Additionally, some natural products were also developed due to the interaction between natural products and genes, such as gelatin, chitosan and polyphenols 16,23,24. Guo et al. described a hierarchical coating (chitosan/gelatin) through the assembly of siRNA-loaded nanoparticles on titanium implants for the synergistic regeneration of skeletal and vascular tissues. The functionalized nanoparticles successfully delivered siRNA cathepsin K (siRNA-CTSK) to bone and showed a high gene silencing efficiency and therapeutic improvement on osteointegration through synergetic effects on bone regeneration and blood vessel system repair 25. However, the existing nucleic acid drug delivery systems are not perfect. For example, the most commonly used cationic liposomes require expensive excipients and equipment for their preparation, thus resulting in high application costs, and they can also produce high toxicity when a high dose is used 26,27. Another obvious disadvantage of liposome preparation is the lack of targeting when administered intravenously, which can lead to adverse reactions, such as hand-foot syndrome 28,29. Besides, polymer-mediated drug delivery systems, such as polyethylenimine (PEI), have a risk of cytotoxicity, poor molecular weight (MW) control, and overly slow or fast degradation kinetics 9,30,31. Inorganic materials are easy to be accumulated in the body and difficult to exclude 32. Therefore, developing new nucleic acid delivery systems with high efficiency and low toxicity has become an urgent strategy in the field of nucleic acid delivery.

**Figure 1:**
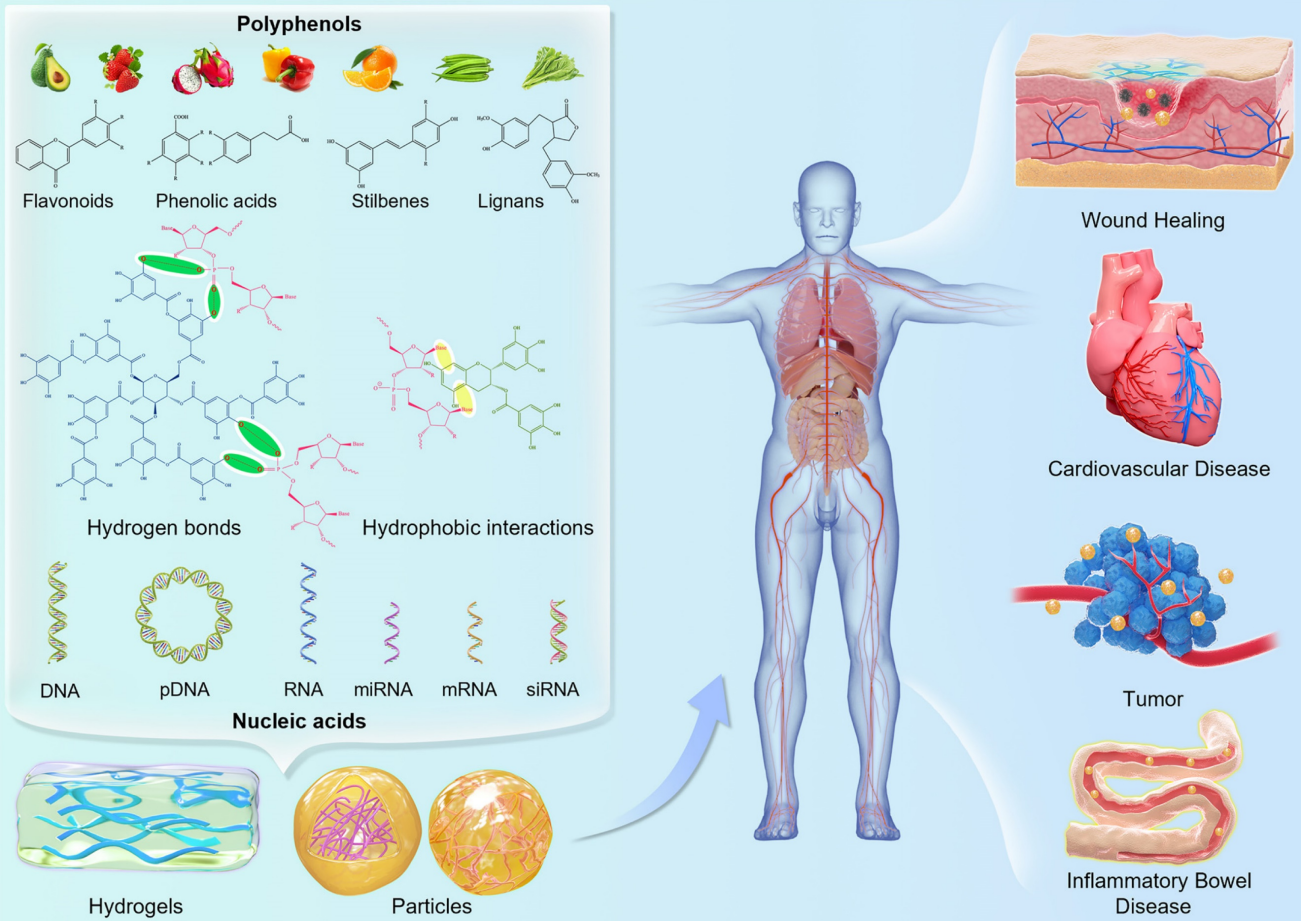
Schematic illustration of the molecular interactions of dietary polyphenols as structural units with nucleic acids for the construction of gene delivery systems in various applications.

Among the myriad of natural product classes, polyphenols are featured by multiple phenolic rings in their basic structure 33,34. Over 8000 phenolics and their derivatives have been identified in various plants, especially in fruits, vegetables, tea, coffees, wine, and grains, which play a role in oxidative stability and sensory properties of food (like flavor, color, bitterness and astringency) 35-37. Polyphenols can be classified into four categories, namely flavonoids, stilbenes, phenolic acids and lignans, according to the number and binding structure of phenolic rings 38. Among them, flavonoids are the most abundant found ubiquitous *in planta*, such as quercetin, epicatechin gallate (ECG), epigallocatechin gallate (EGCG) and catechin (CAT). Benefiting from their fascinating biological activities, such as anti-oxidant, anti-cancer, anti-bacterial, anti-inflammatory, anti-viral, and myocardial protection effects, natural polyphenols have been employed in the field of food, pharmaceutical industries as natural therapeutic agents in recent years 39-52. Besides serving as auxiliary therapeutic drugs, polyphenols can also act as primary construction modules for multiple functional nanomaterials, due to their special structure being rich in benzene rings and hydroxyl groups. It has been demonstrated that natural polyphenols can interact with various materials (small molecules, metal ions, polymers, nucleic acids and proteins) via hydrogen bonding, hydrophobic interactions, π interactions, metal coordination, electrostatic interactions, and covalent bonding 24,53,54. Polyphenol-based functional materials are promising for the development of adhesive materials, surface coating, hemostatic applications, and hydrogels 55-61. Polyphenols have also been functionalized on the surface of living cells via metal-phenolic complex-mediated interfacial interactions to create a versatile cell-based biological platform, in which extrinsic bioactive molecules (proteins, DNA, mRNA) can be imparted to the cells 62. More importantly, they can bind with genes due to their multi-hydroxyl structure, thus making them to be promising and excellent gene delivery vectors. With the employment of polyphenols in innovative delivery strategies, the application of polyphenols in gene delivery systems has been extensively investigated for the reliable nucleic acid delivery. At present, polyphenols have been applied to the construction of delivery systems for a variety of gene therapies, including DNA, siRNA, miRNA, dsRNA, and mRNA (Figure 1). Since polyphenols can complex with nucleic acids to produce nanostructures, which replace the loose state of nucleic acid drugs, thereby reducing the risks of nucleic acid degradation *in vitro* 59. Meanwhile, polyphenols are desired to down-regulate the molecular weight and dosage of cationic polymers under the same conditions, thereby improving the safety of drug delivery systems.

In this review, the most updated status of polyphenols-assisted gene delivery system is summarized and the functional roles of natural polyphenols in the design of gene delivery strategies are discussed. The description of the preparations of different polyphenol-based carriers is briefly reported, while the main part of this review discusses the efficacy of the polyphenols in promoting nucleic acids delivery and gene silencing for disease therapies. In addition, the challenges of polyphenol-based gene delivery systems and prospects for the design of future natural polyphenol drug delivery systems are discussed. Therefore, this review aims to present the current development of polyphenol-assisted gene delivery strategies, thus providing an easily accessible reference and promoting the application of polyphenol-assisted gene delivery systems for researchers and pharmaceutical companies.

## 2. The binding mechanisms of polyphenols and nucleic acids

Nucleic acid is a biological macromolecular compound, which is polymerized from many nucleotide monomers and plays a role as a carrier of genetic information in the life system. There are also some nucleic acids that can be used as enzyme molecules or other molecular machines with biological activities, including ribozymes, deoxyribonucleases, and their complexes. Nucleotide is the basic unit of nucleic acids. A nucleotide molecule is composed of a nitrogenous base, a five-carbon sugar, and a phosphoric acid. With the condensation of phosphoric acids and five-carbon sugars to form the polymerization skeleton of nucleic acids, the phosphate backbone containing dense lone pair electron oxygen is constructed. Notably, the main characteristics of polyphenols as building blocks for drug delivery are the dense *m*-hydroxyl and *o*-hydroxyl groups in the trihydroxy phenyl (galloyl) and dihydroxy-phenyl (catechol) components, such as tannic acid (TA), CAT, and EGCG. The dense phenolic hydroxyl groups represent excellent electron donor groups, which can form intermolecular complexation with the phosphate skeleton containing dense lone pair electron oxygen via intermolecular hydrogen bonds. Besides, nucleic acid molecules contain plenty of bases whose water solubility is unsatisfactory. The abundant benzene rings in polyphenols constitute water-insoluble structure. Through hydrophobic interaction, the benzene ring structure in polyphenols can interact appropriately with the bases in nucleic acids, thus improving the interaction between polyphenols and nucleic acids. Zheng et al. investigated the interaction between EGCG and DNA using electrochemical techniques, and showed that EGCG could intercalate into DNA strands and form an electrochemically inactive complex 63. Fujiki et al. found that single-strand 18 mers of DNA or RNA could bind to 1 to 3 EGCG molecules in surface plasmon resonance assay (Biacore) and cold spray ionization-mass spectrometry, suggesting that multiple binding sites of EGCG were present in DNA and RNA oligomers 64. Using enhanced sampling techniques and molecular dynamics simulations, Rodrigo et al. observed that both benzopyran moiety ring and trihydroxyphenyl ring of EGCG could form hydrogen bonds with the oxygen atoms in the DNA backbone of the 5'-strand, and a stable complex was formed between the EGCG ligand and DNA by intercalating the trihydroxybenzoate aromatic ring and an ApC step 65. In summary, the interactions between polyphenols and nucleic acids are synergistically mediated by both intermolecular hydrogen bonds and hydrophobic interactions, which are the basic mechanism of building polyphenol-assisted drug delivery systems.

## 3. Polyphenol-assisted nucleic acid delivery

Nucleic acids, the general name of DNA and RNA, are a kind of biopolymer and the indispensable genetic materials of all creatures. The classification of nucleic acids is mainly determined by the five-carbon sugars involved in construction. If the pentose is ribose, the polymer formed is RNA; if the pentose is deoxyribose, the nucleic acid formed is DNA. As genetic molecules, nucleic acids are excellent candidate polymers for biomaterial applications with precise molecular recognition, superior sequence programmability, and extensive biological functionality 33,66. Successful delivery of genetic material plays an important role in gene therapy, and polyphenols have a strong binding affinity to macromolecular nucleic acids via forming hydrogen bonds, thus increasing their *in vivo* stability, which can be a safe and high-efficient gene vector.

### 3.1 Polyphenol-assisted DNA delivery

**Figure 2:**
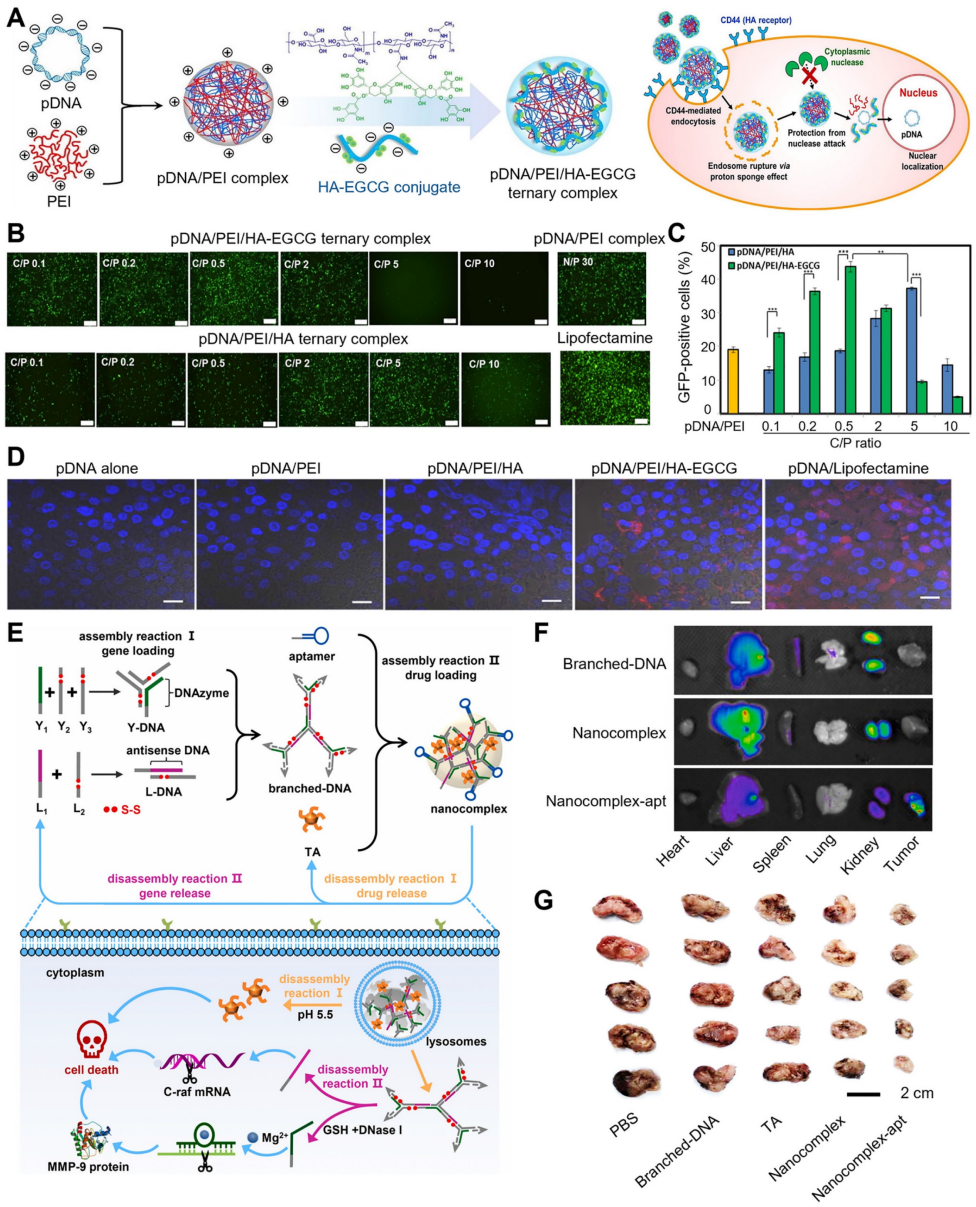
Polyphenol-assisted DNA delivery. (A) The self-assembled formation and cellular uptake process of pDNA/PEI/HA-EGCG ternary complexes. (B) The GFP gene transfection efficiency in HCT-116 cells treated with different complexes at different C/P ratios. (C) Quantification of GFP expression in HCT-116 cells. (D) Intratumoral distribution of Cy5-labeled pDNA delivered by various polyelectrolyte complexes. Adapted with permission from 70, copyright 2016 Elsevier. (E) Schematic illustration of the assembly and controlled disassembly process of DNA nanocomplex in cells. (F) *Ex vivo* biodistribution of DNA at major organs and tumors of the mice treated with different formulations. (G) Tumor size in A549 tumor-bearing mice treated with different formulations. Adapted with permission from 71, copyright 2021 Elsevier.

Various cationic polymers have been employed to condense plasmid DNA (pDNA) into nanoscale complexes via electrostatic interactions, especially PEI, which facilitates endosomal escape and promotes cell internalization 67,68. However, cytotoxicity and agglutination with blood components hinder the clinical application of pDNA/PEI complex 69. Therefore, Liang et al. firstly designed a gene delivery system based on green tea catechin, a nanoscale self-assembled ternary complex (pDNA/PEI/HA-EGCG), which exhibited remarkable protection of pDNA from nuclease hydrolysis and polyanion-induced dissociation and ascertained high transfection efficiency for the difficult-to-transfect HCT-116 cells even under serum-supplemented conditions (Figure 2A-D) 70. In this DNA delivery system, hyaluronic acid (HA) did not only improve gene transfection efficiency through endocytosis mediated by HA receptor, but rather effectively downregulated the potential cytotoxicity and pDNA/PEI agglutination via shielding the surface positive charges. Besides, the natural polyphenol EGCG could stabilize pDNA/PEI complexes owing to its strong DNA-binding affinity and is known to suppress the activity of various enzymes, such as nucleases, collagenases and hyaluronidases via blocking their active sites, thereby protecting DNA against degradation dominated by serum proteases *in vivo*. Two cell lines were employed to evaluate gene transfection efficiency of pDNA/PEI* in vitro*. In HEK293 cells (cluster of differentiation 44 (CD44) low-expressed), the transfection efficiency was 73.8 ± 5.2% with N/P ratio of 30, while the transfection efficiency was 19.0 ± 0.8% in HCT-116 cells (CD44 over-expressed). At the optimal C/P ratio of 0.5, the transfection efficiency of pDNA/PEI/HA-EGCG system was improved to 43.7 ± 1.5% in HCT-116 cells, which was approximately 2.3-fold higher than that of pDNA/PEI (18.6 ± 0.6%). In addition, compared to pDNA alone, pDNA/PEI complexes and pDNA/PEI/HA complex, pDNA/PEI/HA-EGCG was successfully delivered into mouse HCT-116 tumor tissues with significantly increased distribution of pDNA in tumors tissues, suggesting that this HA-EGCG-stabilized system could potentially be utilized in CD44-targeted nucleotide therapeutics delivery.

To co-deliver genes and anti-tumor agents, Yang et al. developed a general and simple carrier based on DNA-polyphenol nanocomplex that is characterized by controllable assembly/disassembly behaviors, thus exhibiting extremely high gene/drug loading capacity (Figure 2E-G) 71. Briefly, two extrinsic genes antisense DNA and DNAzyme were structurally designed as branched-DNA, thus DNAzyme was prepared as Y-shape DNA (Y-DNA) while antisense DNA was programmed into L-DNA. Then, Y-DNA and L-DNA formed branched DNA via hybridization, and TA intervened the assembly of branched-DNA to form nanosized complexes. The encapsulation efficiencies of antisense DNA, DNAzyme, and TA in this nanosystem were at 94.4%, 96.2%, and 85.2%, respectively. To improve the tumor-targeting efficiency, DNA aptamer was effectively anchored onto this nanocomplex (nanocomplex-apt). The acid microenvironment of lysosomes triggered the release of TA and branched-DNA, and branched-DNA was further separated as antisense DNA and DNAzyme by DNase I and glutathione (GSH). This nanocomplex exerted specific cytotoxicity to A549 cells and showed an excellent biocompatibility with normal cells, suggesting its safety of use. Antisense DNA conspicuously inhibited the hyperplasia of A549 cells through reducing C-raf mRNA expression. Besides, TA facilitated cancer cell apoptosis via decreasing the expression levels of apoptosis-related proteins (such as Bcl-2, an anti-apoptosis protein). The incorporation of DNA aptamer up regulated the proliferation inhibition of A549 cells due to the targeting capacity of the aptamer. DNAzyme also inhibited A549 cell migration through reducing matrix metallopeptidase 9 protein (MMP-9) expression, thus further improving the therapeutic efficacy of nanocomplex-apt. In A549 tumor-bearing mice, nanocomplex-apt showed specific targeting to tumor and achieved the best medical intervention effects on cancer cells, which demonstrated the synergistic effects of chemotherapy and multiple-gene therapy.

**Figure 3:**
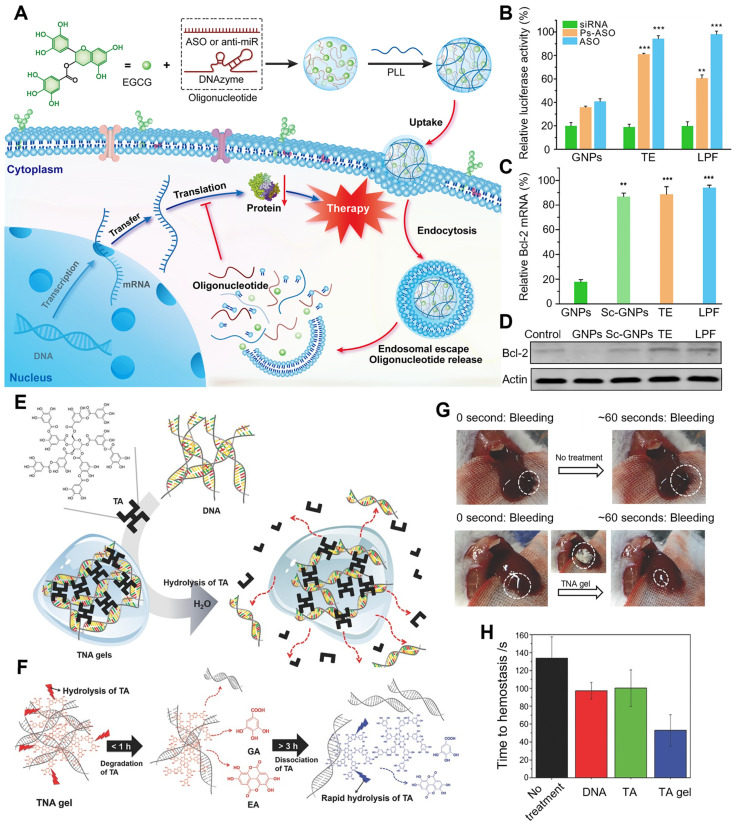
(A) Schematic illustration of GNPs construction and delivery of single-strand oligonucleotides including anti-miRNA, ASO, and DNAzyme. (B) Efficiency of GNPs in the delivery of siRNA, Ps-ASO, and ASO into Hela-Luc cells for 24 h. (C) The mRNA expression levels of Bcl-2 in Hela cells after treatment with GNPs loaded with DNAzyme targeting Bcl-2. (D) Representative Bcl-2 protein expressions in treated cells. Adapted with permission from 72, copyright 2020 Springer Nature. (E) Schematic illustration of the formation and degradation of TNA gels. (F) The degradation process of TNA gels. (G) Images of the hemostatic effect of TNA gels within 60 s. (H) The required times for complete hemostasis of the bleeding mouse liver. Adapted with permission from 73, copyright 2015 WILEY.

Cheng et al. developed a facile, robust, and efficient strategy in delivering single-strand oligonucleotides (Figure 3A-D) 72. The natural polyphenol EGCG was employed to combine with oligonucleotide to prepare a negatively charged core, then ε-polylysine (PLL) (positive charge) was covered to the surface of EGCG/oligonucleotide to form the core-shell nanostructure, which was termed as green nanoparticles (GNPs). Next, three different oligonucleotides were chosen to estimate the application potential of GNPs in gene delivery, including miRNAs, antisense oligonucleotide (ASO), and DNAzymes. For delivering ASO, GNPs protected ASO from RNases degradation, which might attribute to the core-shell structure of GNPs. GNPs, Lipofectamine 2000 (LPF) and TransExcellent-siRNA (TE), exhibited prominent gene-silencing efficiency in delivering siRNA. However, compared to LPF and TE, GNPs showed higher gene knockdown efficiency in delivering unmodified ASO (59%) and phosphorothioate backbone ASO (Ps-ASO) (64%), due to the good stability of oligonucleotides in GNPs and efficacious endocytosis and intracellular release. The successful delivery of DNAzymes could target the oncogene Bcl-2 in Hela cells and downregulate the expression of Bcl-2 mRNA, thus effectively inhibiting Bcl-2 protein expression and increasing apoptosis.

Besides, polyphenol-based hydrogels are also widely used in innovative drug delivery systems. TA, a natural polyphenol, is often utilized as a natural anti-inflammatory, anti-oxidant, and antibacterial agent. More importantly, it is also an exceptional crosslinking agent for the preparation of innovative hydrogel delivery system due to its high content of natural catechol/pyrogallol moieties. Lee et al. utilized TA as a molecular glue to produce a DNA hydrogel, called TNA hydrogel, whose crosslinking mechanism was the formation of hydrogen bonds between DNA's phosphate backbone and TA's polyphenol (Figure 3E-H) 73. At a stoichiometric ratio of DNA base pairs/TA of 1.3, spontaneous TNA gelation was observed after TA was added into the DNA solution, while no gelation was occurred when the ratio was at 2.6. In contrast, mechanically robust TNA gels were formed when the ratio was downsized from 1.3 to 0.9. The TNA gel was degradable due to the hydrolysable ester bonds between the catechol and pyrogallol groups in TA, which resulted in the release of DNA from the gel. The dissociation and degradation of TNA gel were able to be divided into two steps. In the first step (0.5-1h), TA in this gel was degraded to ellagic acid (EA) and gallic acid (GA) at low extent owing to the steric hindrance of the complexed DNA. After > 3h, TA was dissociated from TA/DNA complexes and then degraded, thereby increasing the amounts of free EA and GA, which was accompanied with more DNA release. With excellent extensibility, biocompatibility and strong mechanical properties, TNA gel displayed biomedical potential as controllable adhesives. In a mouse liver bleeding model, TNA gel exhibited an excellent hemostatic effect and obviously shortened the hemostatic time when compared with negative control and single-component solutions, demonstrating that hemostasis was enhanced by the adhesive property of TNA gel. With biodegradability, extensibility, tissue adhesiveness and hemostatic ability, this multifunctional TNA gel could be an innovative DNA-based platform for future biopharmaceutical applications.

### 3.2 Polyphenol-assisted RNA delivery

RNA interference (RNAi) can specifically down-regulate target genes by delivering small RNA duplexes, including siRNAs, miRNA mimics, dicer substrate RNAs (dsiRNAs) and short hairpin RNAs (shRNAs), which provides alternative treatment options for various diseases when current drug fails 74-76.

#### 3.2.1 Polyphenol-based siRNA delivery

Gene therapy via siRNA has been a promising technique for the treatment of cancer and other diseases by preventing the target proteins production 77. However, naked siRNA is unstable in physiological environment and susceptible to enzyme degradation, and the highly negative charge limits its penetration through the cell membrane 78. Thus, successful RNAi therapies are heavily dependent on the construction of efficient gene delivery platforms.

**Figure 4:**
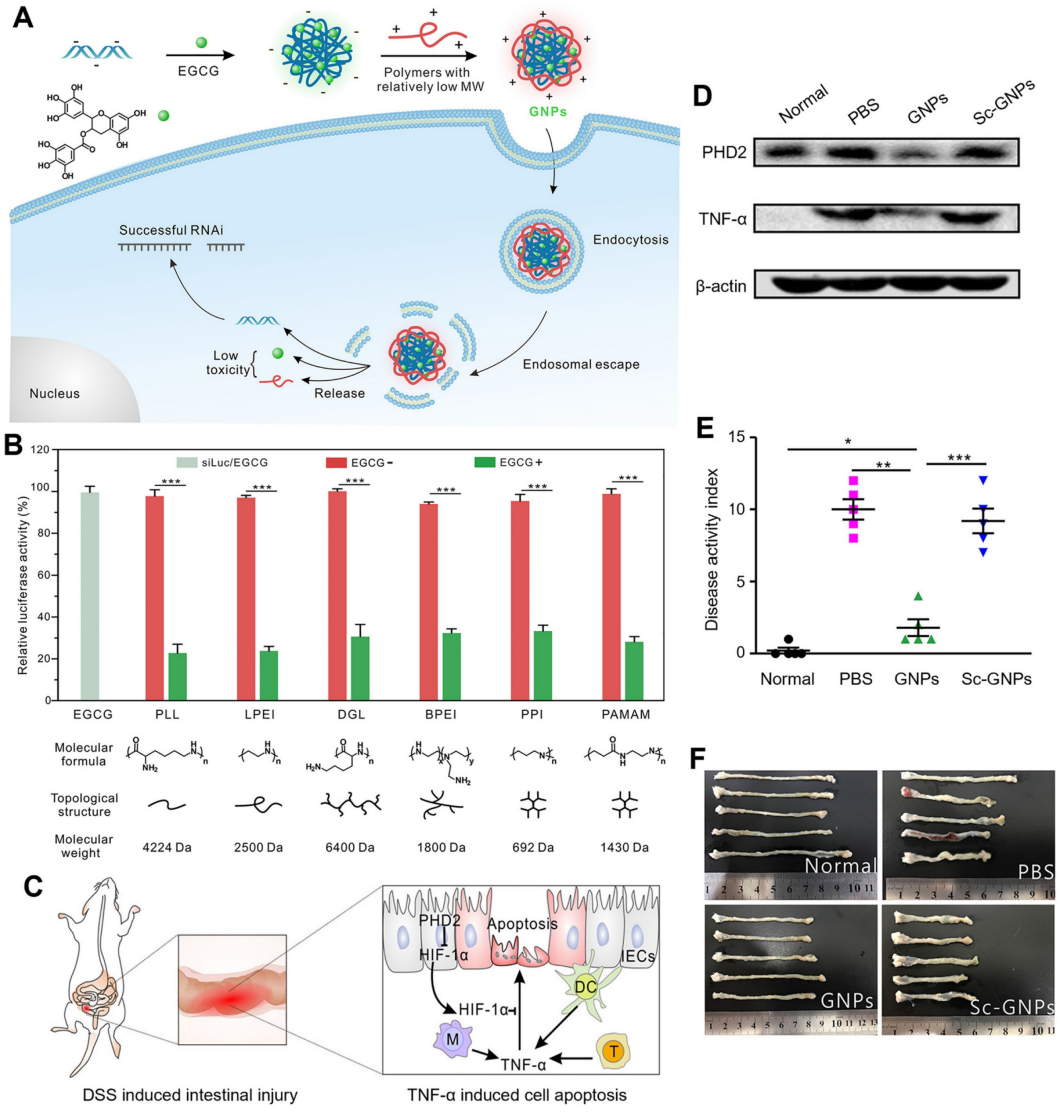
Polyphenol-based RNAi efficient delivery. (A) Schematic formation and intracellular gene silencing mechanism of GNPs. (B) Different polymers mediated RNAi efficiency in HeLa-Luc cells for 24 h. (C) The mechanism of TNF-α induced cell apoptosis in DSS-induced intestinal injury model. (D) The protein levels of PHD2 and TNF-α in the colon tissues. (E) Disease activity index and (F) colon length of mice after different treatments. Adapted with permission from 79, copyright 2018 American Chemical Society.

Cheng et al. designed a simple and general supramolecular strategy to fabricate the core-shell nanostructure for siRNA delivery, in which siRNA was pre-complexed with EGCG as the negatively charged core and then coated with low-molecular-weight cationic polymer as the shell (Figure 4A-F) 79. EGCG, that is complexed with siRNA via hydrogen bonds and hydrophobic interactions, largely improved the siRNA complexation ability of low-molecular-weight polymers. The addition of EGCG facilitated the formation of smaller, more uniform and stable GNPs, which showed a good stability in 150 mM sodium chloride (NaCl) solution and cell culture medium. In addition, six types of different molecular weight cationic polymers (including linear, branched, and dendritic polymers) were tested to balance the transfection efficiency-toxicity in siRNA delivery. Compared with minimal toxic low-molecular-weight cationic polymers, the high-molecular-weight ones exhibited high efficiency but possessed serious toxicity. In contrast, both low toxicity and high transfection efficiency were achieved, when GNPs were fabricated with these 6 screened low-molecular-weight polymers. In HeLa cells with stable expression of firefly luciferase, all chosen GNPs exhibited high gene silencing efficiencies (~80%) compared with the nanoparticles without EGCG (< 5%). Among these 6 representative cationic polymers, *ε*-PLL was preferred for fabricating GNPs for the *in vivo* experiments due to the high siRNA delivery efficiency and good biocompatibility. In a dextran sulfate sodium (DSS)-induced mouse intestinal injury model, GNPs exhibited a distinct reduction of prolyl hydroxylase 2 (PHD2) and TNF-*α* gene expressions in the colonic tissues. Compared to control, GNPs effectively ameliorated intestinal symptoms and inflammation, including lowered disease activity index (DAI) score, less body weight loss, shortened colon length and reduced inflammatory cytokines levels. All these results indicated that this supramolecular strategy of the construction of GNPs can be a versatile and potential method for various gene delivery. In addition to cationic polymers that could condense with siRNA, different polyphenol moieties can also affect the binding and deliverable capacity of siRNA. The structure-function relationship of polyphenolic moieties in facilitating siRNA condensation and delivery was further investigated as the natural polyphenol EGCG could be hydrolyzed into two other polyphenol motifs, EGC and GA 80. Their results revealed that EGCG and EGC exhibited enhanced binding ability to siRNA through hydrogen bonds and hydrophobic interactions, while GA with a pyrogallol structure could hardly bind to siRNA and suggestive that it does not play a role in binding to nucleic acids. In the experiment of polyphenol assisted PLL delivery of siRNA targeting luciferase (siLuc), the delivery capacity was in the following order EGCG > EGC > GA, with corresponding gene silencing efficiency of 80%, 40%, and almost zero, respectively. As the molar ratio of EGC/EGCG increased, the gene knockdown efficiency was significantly increased, which provided novel insights for the search for other natural polyphenols with similar chemical structures to effectively assist gene delivery and further provided theoretical support for the development of more safe and efficient RNAi drugs.

**Figure 5:**
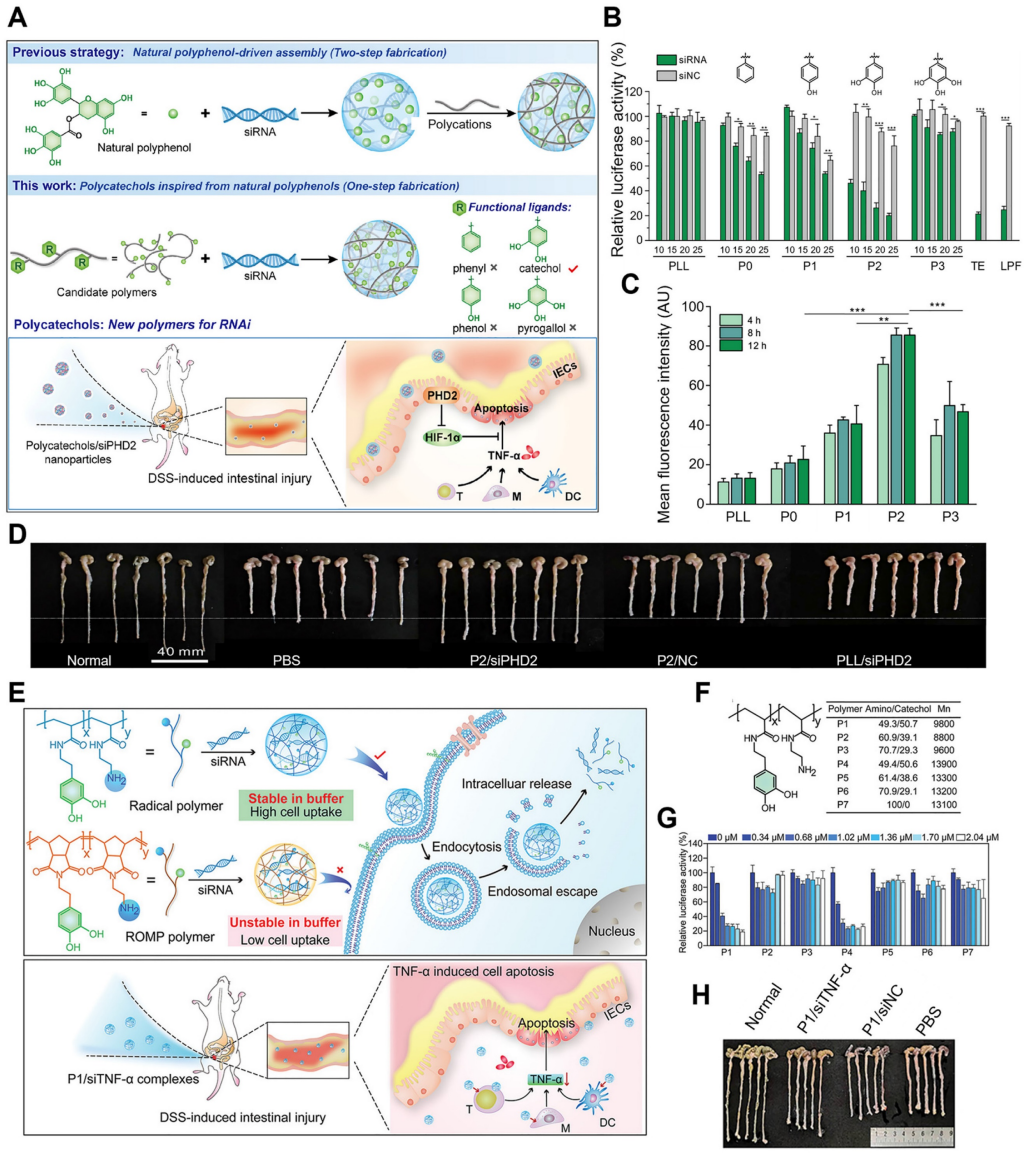
Polyphenols with different structures for siRNA delivery. (A) Schematic illustration of the synthetic route of polycatechols with different phenolic moieties for siRNA delivery. (B) Gene silencing efficiency of PLL and P0-P3 in delivering siLuc to HeLa-Luc cells. (C) Cellular uptake efficiency of polymer/siRNA-FAM complexes in HeLa-Luc cells. (D) Protein expression levels of PHD2, HIF-1*α*, and TNF-*α* in colon tissues analyzed by Western blotting. (E) Colon length of the mice in different groups after treatment. Adapted with permission from 81, copyright 2021 WILEY. (F) Schematic illustration of the synthesis method of the polymers and the gene silencing mechanism of siRNA delivery in cells and DSS-induced intestinal injury mice. (G) The structures, molecular weights of polymers P1 - P7 (synthesized by radical polymerization) and polymers P8 - P10 (synthesized by ROMP). (H) The luciferase gene knockdown efficiencies of polymer/siLuc (P1 - P7) in HeLa-Luc cells for 24 h. (I) Colon length of mice after treatments. Adapted with permission from 82, copyright 2020 Chinese Chemical Society.

Inspired by the structural and functional characteristics of natural polyphenols, a novel strategy to simplify the synthesis process into one step was attempted, in which the functional moieties from naturally occurring polyphenols were first grafted onto low-molecular-weight cationic polymers to fabricate poly-catechol polymers, thus improving siRNA binding ability (Figure 5A-E) 81. The phenyl, phenol, catechol, and pyrogallol-modified PLL polymers were synthesized and termed P0-P3 separately, then the corresponding polymer/siRNA complexes were prepared. P2, with catechol groups showed the highest luciferase gene silencing efficiency, albeit with certain toxicity in HeLa-Luc cells. Besides, in HeLa and mouse intestinal epithelial cells (IECs), P2/siRNA complex successfully downregulated its targeted glyceraldehyde 3-phosphate dehydrogenase (GAPDH) and PHD2 gene expression. The gene silencing efficiency was related to the siRNA binding ability of polymers in the order of P2 > P1 > P3 ≈ P1 > PLL, which was due to the balance of hydrophobic interactions and hydrogen bonding between siRNA and polyphenols. As the number of hydroxyl groups on the aromatic ring increased, hydrophobic interactions were weakened while hydrogen bonds became more dominant. Besides, the interruption of the balance of hydrogen bonding and hydrophobic interactions resulted in a decrease of the affinity of siRNA for the polymer. In DSS-induced intestinal injury or colitis model, P2/siRNA successfully delivered siRNA to the injured tissues and significantly relieved the symptoms and reduced inflammatory levels. Inspired by these results, Fan et al. also designed and synthesized a set of cationic poly-catechols for siRNA delivery by two different direct polymerization methods. Several important macromolecular parameters affecting siRNA delivery efficiency were investigated, such as catechol content, molecular weight, and backbone rigidity (Figure 5F-I) 82. Poly-catechols P1 - P6 were fabricated via radical polymerization, whereas P8 - P18 were via typical ring opening metathesis polymerization (ROMP) method. Furthermore, polymers were complexed with siLuc. Polymers P1 and P4 of high catechol contents (50%) potently downregulated luciferase gene expression and exhibited better performance in siRNA condensation, protection, and cellular uptake, while polymers with lower catechol contents (40% for P2 and P5; 30% for P3 and P6) failed to silence luciferase gene at the same molar concentrations. P8 - P18, with more rigid backbones, showed poor efficiencies in siRNA delivery, which revealed that catechol-derived polymers with high catechol content and flexible backbones (P1 and P4) were the best for efficient siRNA delivery. In addition, compared with P4, P1 still maintained high gene knockdown efficiency even at a low siRNA dose, and showed high siRNA efficiency and efficiently downregulated TNF-α gene expression. In DSS-induced ulcerative colitis (UC) model, P1/siTNF-α effectively downregulated TNF-α level and ameliorated UC symptoms without any adverse effects.

Therefore, this study provided strong support for the synthesis and screening of polyphenols for siRNA delivery. In addition to gene delivery, the synthesized polycatechols were also utilized as a promising drug delivery platform for cytosolic protein and peptide delivery, which greatly promoted the delivery efficiency 83,84.

**Figure 6:**
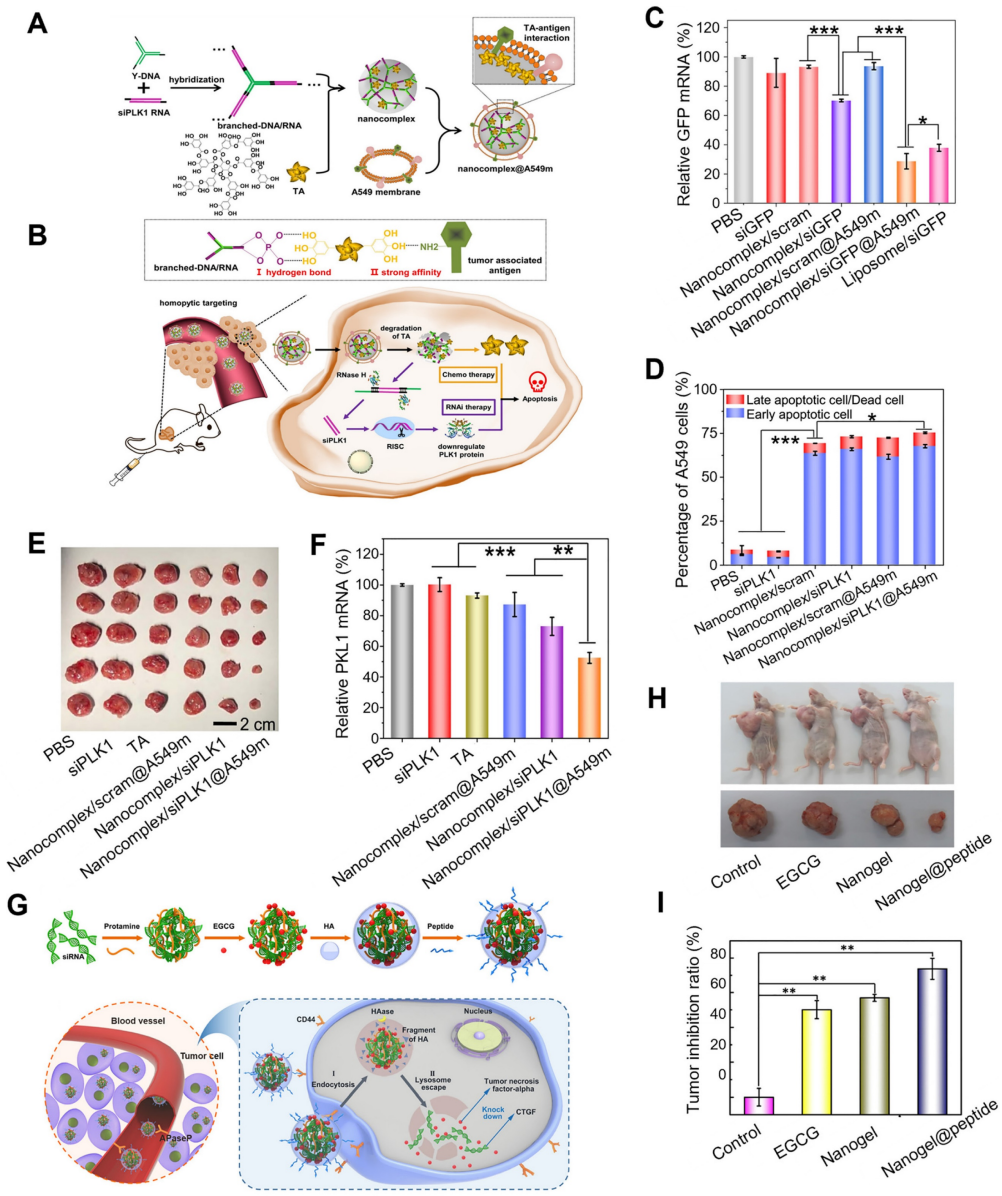
Polyphenol assisted siRNA delivery. (A) Schematic construction of nanocomplex@A549m. (B) Scheme of the synthesis and controlled disassembly of the nanocomplex. (C) The expression level of relative GFP mRNA in A549-EGFP cells analyzed by qRT-PCR. (D) The apoptotic percentages of A549 cells. (E) The dissected tumors after treatment. (F) The expression levels of PLK1 mRNA in tumor-bearing mice. Adapted with permission from 85, copyright 2021 Elsevier. (G) Schematic illustration of the self-assembly of nanogel/peptide and the enhanced treatment of CTGF-overexpressing TNBC. (H) The antitumor activity of the nanogel in MDA-MB-231 tumor-bearing mice after different treatments. (I) Corresponding tumor inhibition ratio of different formulations. Adapted with permission from 86, copyright 2018 American Chemical Society.

Considering the dual roles as the structural units and therapeutic agents, polyphenols are used in combination therapy of gene cancer therapies. Yang et al. utilized natural TA as a “sandwich component” that binds to nucleic acids and tumor-associated-antigen of cancer cell membranes, thus constructing a smart system for drug delivery and gene therapy that delivered therapeutic RNA to target cells (Figure 6A-F) 85. Firstly, the sticky ends of siRNA were attached to the complementary sticky ends of Y-DNA to form branched-DNA/RNA, TA was then added to mediate both assemblies of branched-DNA/RNA and A549 cell membrane to form a nanocomplex (nanocomplex@A549m), which could mediate the dissociation of the nanocomplex in the lysosomal acidic microenvironment and exerted pro-apoptotic effects on cancer cells. In A549 cells, nanocomplex@A549m showed high cellular uptake efficacy, specific homotypic targeting capability and reduced macrophage cell internalization. Furthermore, in A549 tumor-bearing mouse model, nanocomplex/siPKL1@A549m effectively delivered therapeutic siRNA to tumor sites and exhibited high RNAi efficiency and enhanced anti-tumor activity, which were also confirmed by *in vitro* assays. Zhu et al. described a high-effective and low-toxic multi-component vector, that was capable of facilitating gene and drug co-delivery for the treatment of drug-resistant breast cancer overexpressing connective tissue growth factor (CTGF), which encapsulated both EGCG and siRNA into a biodegradable nanogel through a self-assembly process (Figure 6G-I) 86. In this biodegradable system, positively charged protamine was employed as the “adhesive” to encapsulate sufficient amounts of siRNA and EGCG, and the outer surface was coated with HA and cell-penetrating peptide PEGA-pVEC. In this system, HA coating could specifically identify the overexpressed CD44 receptor on the surface of cells to induce endocytosis mediated by clathrin and triggered EGCG release by hyaluronidase (HAase), while PEGA-pVEC could target the proline aminopeptidase overexpressing in the blood vessels of breast tumor. Compared to CTGF-negative MCF-7 cells, siRNA/EGCG/protamine/HA nanogel showed high selectivity to CTGF-overexpressed MDA-MB-231 cells, due to the specific recognition of CD 44 receptor and endogenous HAase-induced drug release. Besides, compared to EGCG, siRNA/EGCG/protamine/HA showed a 15-fold increase in cytotoxicity against drug-resistant MDA-MB-231 cells, which was attributed to the synergistic effect of EGCG and siRNA. In MDA-MB-231 xenograft tumor model, nanogel@peptide displayed the highest tumor inhibition effect and little toxicity to normal tissues and organs. Additionally, the expressions of cellular apoptosis protection related proteins (phosphor-focal adhesion kinase (p-FAK), phospho-extracellular regulated protein kinases (p-ERK), and poly ADP-ribose polymerase (PARP)) and drug resistance related proteins (B-cell lymphoma-extra large (Bcl-Xl), cellular inhibitor of apoptosis protein 1 (cIAP1), and CTGF) were down-regulated both *in vitro* and *in vivo*.

Natural polyphenols have also been utilized as functional coatings to assist gene delivery. Recently, a novel type of core-shell structured MSN (MNC@LPMSA@siRNA@TA) with large pore was proposed for siRNA delivery, which possessed small particle sizes, high siRNA loading capacity, magnetic-guided delivery ability and pH-responsive cellular siRNA release 87. The magnetic nanocrystal clusters (MNC) were firstly synthesized and coated with dendritic mesoporous silica layer to yield core-shell MSC@LPMS, which were then functionalized with (3-aminopropyl)-triethoxysilane (APTES) to obtain a positively charged silica surface for the loading of negatively charged siRNA (loading efficiency ca. 2% w.t.) due to the high pore volume and surface area. Finally, TA/Al^3+^ complex, acid-resistant coating formed via pH-dependent complexation, was coated to MNC@LPMSA@siRNA to protect siRNA against degradation, thereby improving the stability of these nanoparticles. These nanoparticles were then endocytosed and entered the acidic lysosomes through the tight binding between Al^3+^ and the cell membrane. Then TA/Al^3+^ coating was disassembled in the acidic environment of lysosome, which consumed protons (H^+^) and facilitated intracellular endosomal escape through the “proton sponge” effect. In osteosarcoma KHOS cells, siRNA was effectively delivered into the cytoplasmic region and under the magnetic field, it showed the highest cellular uptake, with negligible cytotoxicity.

Metal-polyphenol networks (MPNs) is an increasingly attractive topic that is used in gene delivery in recent years. Chen et al. developed a green carrier (Mg(II)-Cat NPs) for efficient siRNA delivery through the chelation of natural polyphenol ((+)-catechin) with metal ions (Mg^2+^) 88. Mg(II)-Cat NPs were prepared by the reaction of Mg^2+^ with adjacent hydroxyl groups on catechin at room temperature. siEIF5A2 was chosen for targeting and knocking down the oncogene eukaryotic translation initiation factor 5A2 (EIF5A2), thus inhibiting cancer cell growth. How would be the enantiomer of catechin? i.e., epicatechin that performs in siRNA delivery and its effect of stereochemistry on nucleic acid binding has yet to be fully explored. In T24 cells, Mg(II)-Cat/siEIF5A2 successfully down-regulated the gene expression of EIF5A2 and induced cancer cell apoptosis. In athymic mice bearing T24 cells xenograft model, Mg(II)-Cat/siEIF5A2 was effectively accumulated in tumors and exerted significant antitumor effects via inhibiting phosphoinositide 3-kinase/ protein kinase B (P13K/Akt) signaling pathway. In addition, Mg(II)-Cat/siEIF5A2 was also found to be an active anti-tumor agent in a clinically-relevant rat in-situ bladder cancer model, suggesting that metal-polyphenol network was a promising platform for the co-delivery of siRNA and chemotherapeutic agents.

**Figure 7:**
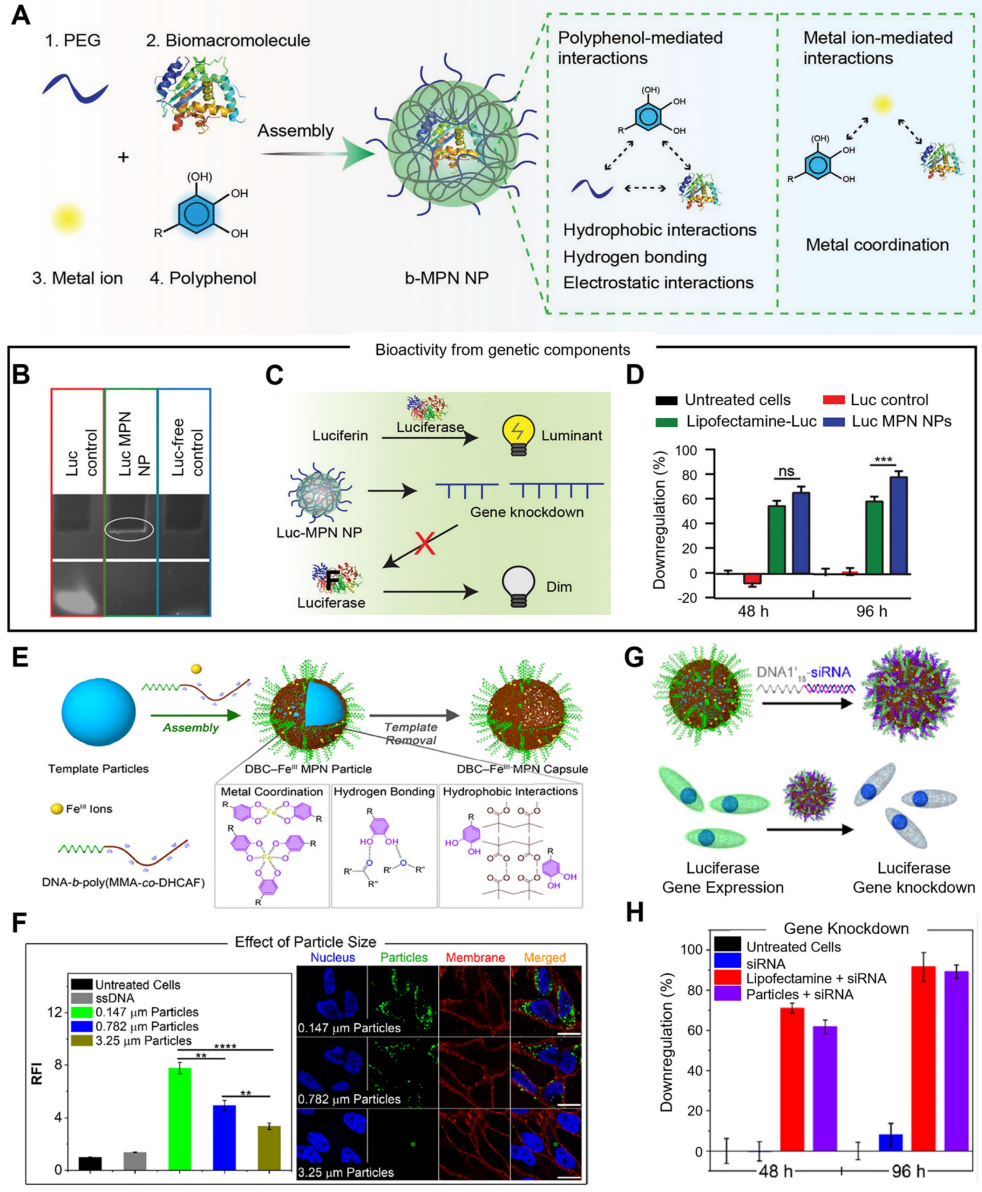
Metal-phenolic networks for siRNA delivery. (A) Schematic illustration of the assembly of b-MPN NPs driven by metal-phenolic interactions. (B) TBE polyacrylamide gel electrophoresis of free Luc siRNA (Luc control), Luc-MPN NPs, and Luc-free NPs (Luc-free control). (C) Schematic illustration of luciferase siRNA working mechanisms. (D) The luciferase gene silencing efficiency in PC3-Luc2 cells at 48 and 96 h after transfection. Adapted with permission from 89, copyright 2022 WILEY. (E) Schematic illustration of the preparation of DBC-Fe^III^ MPN particles and capsules through the assembly of catechol-modified DBC and Fe^III^ ions. (F) Quantitative and qualitative analysis of the cellular uptake of different sized DBC-Fe^III^ particles after 24 h. (G) Schematic illustration of the preparation of siRNA-functionalized DBC-Fe^III^ MPN particles and working mechanism. (H) Luciferase gene knockdown efficiency in PC3-Luc2 cells after 48 and 96 h transfections. Adapted with permission from 90, copyright 2022 American Chemical Society.

In addition, Caruso et al. designed a metal-phenolic assembly approach to fabricate bioactive metal-polyphonic nanoparticles (b-MPN NPs) via the one-pot assembly of biomacromolecules, metal ions, and polyphenols nanoparticles, which was driven by metal-polyphenol coordination, hydrogen bonding and hydrophobic interactions (Figure 7A-D) 89. Poly(ethylene glycol) (PEG) was acted as the seeding agent that could locally increase the concentrations of the metal ion, phenolic ligand, and biomacromolecule precursors. Various phenolic building blocks (i.e., EGCG, CAT, GA and TA), and metal ions with different valences and coordination states (i.e., Cu^II^, Fe^III^, Al^III^, Zr^IV^, and Ti^IV^) could be employed to b-MPN NP platform, demonstrating its tunability and versatility. In the delivery of siRNA, EGCG and Zr^IV^ were selected as the phenolic ligand and metal ion, respectively, and luciferase siRNA was assembled into the nanoparticles (Luc-MPN NPs), which was proved by 10% tris-borate-EDTA (TBE) polyacrylamide gel electrophoresis. Luc siRNA was successfully delivered into PC3 cells expressing the firefly luciferase gene (PC3-Luc2), and 80% of luciferase gene expression was downregulated by Luc-MPN NPs at up to 96 h after transfection, which was comparable to the commercial cationic lipid-mediated transfection agent Lipofectamine RNAiMax (Lipofectamine-Luc). Due to the multi-choice of metal ions, phenolic ligands and biomacromolecules, this system was performed in a range of applications, such as delivering cytochrome C for cell apoptosis, RNase A for RNA degradation, and glucose oxidase/catalase co-assembly complex for toxic intermediates elimination via cascade reactions. Then, they synthesized DNA-functionalized metal-phenolic networks through the assembly of catechol modified DNA block copolymer (DBC) and metal ions, in which assembly process was driven by the electrostatic interactions between phenolic groups and metal ions (Figure 7E-H) 90. Catechol-functionalized DBC (DNA-*b*-poly(methyl methacrylate-co-2-methacryloylethyl dihydrocaffeate, DNA-*b*-poly(MMA-*co*-DHCAF)), that was composed of DNA segment, catechol group and hydrophobic group, was used as a building block and exhibited molecular recognition properties of DNA. Fe^III^ was exploited as the metal ion to construct DBC-based MPN nanoparticles and capsules (DBC-Fe^III^ capsules), which could be stably existed in acidic, metal-chelating, and surfactant solutions due to the multiple assembly interactions (metal coordination, hydrogen bonding and hydrophobic interactions). In HeLa cells, the cellular uptake of DBC-Fe^III^ MPN particles was increased when the particle size was decreased, as smaller particles possessed more dense DNA strands on the surface, suggesting that high DNA surface density is beneficial for cell internalization of DBC-Fe^III^ MPN particles. Therefore, DBC-Fe^III^ MPN particles with a size of 0.147 μm were used in a subsequent study. Luciferase siRNA-functionalized DBC-Fe^III^ MPN particles were designed through conjugating siRNA to the complementary sequence of DNA1 (DNA1'_18_-siRNA) for the delivery of siRNA. In PC3-Luc2 cells, 89% of the luciferase gene was silenced by siRNA-functionalized DBC-Fe^III^ MPN nanoparticles, while the free siRNA strands had no effect on gene silencing, demonstrating that DNA-functionalized metal-phenolic systems were potentially biocompatible nucleic acid delivery vehicles.

#### 3.2.2 Polyphenol-assisted miRNA delivery

**Figure 8:**
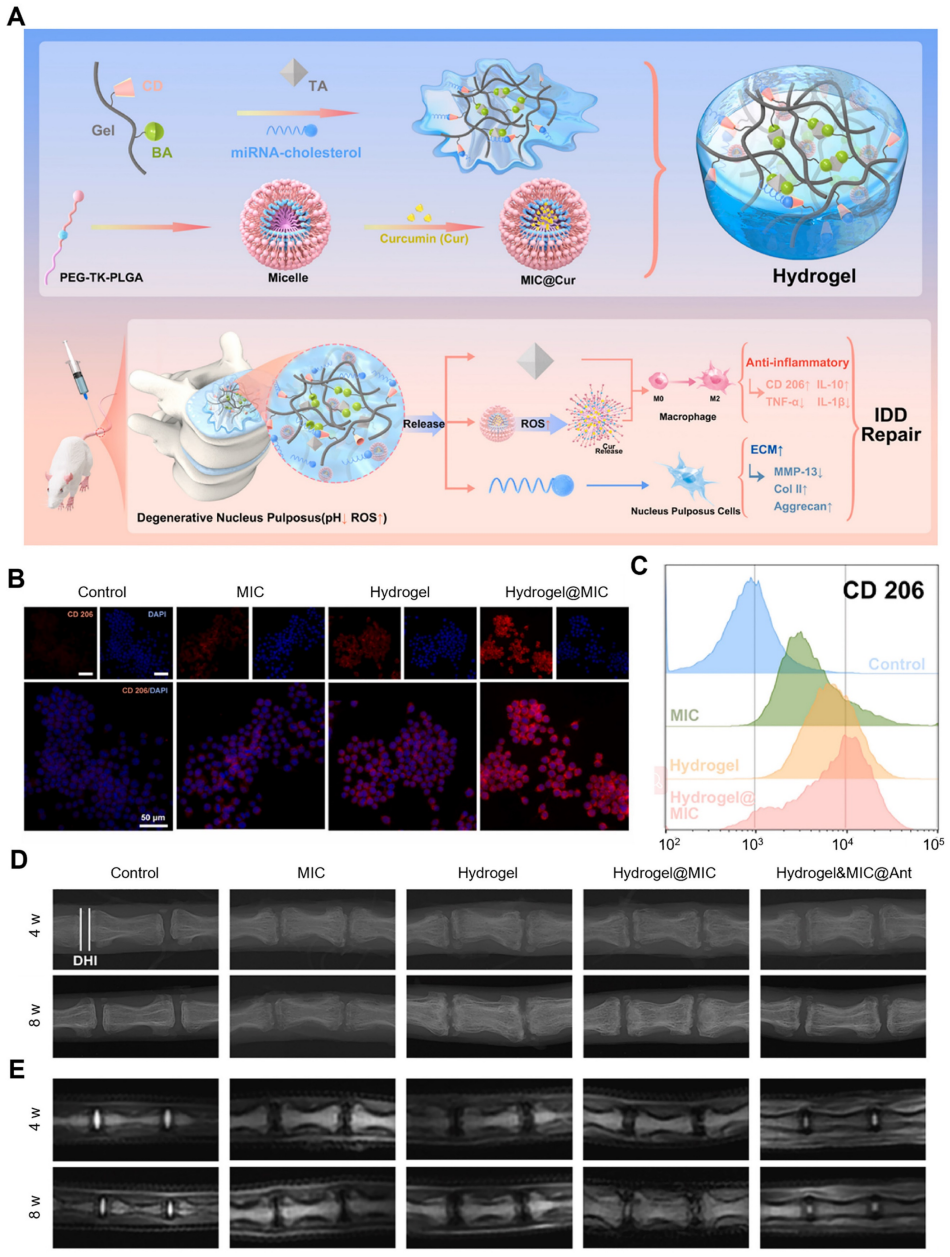
Polyphenol-assisted miRNA delivery. (A) Schematic illustration of the formation of inflammation-responsive hydrogels and the mechanisms of facilitating IDD repair. CD206 expression levels in different groups analyzed by (B) immunofluorescence staining and (C) flow cytometry. (D) X-ray and (E) MRI images of rat coccygeal vertebral discs in different groups at 4 and 8 weeks. Adapted with permission from 91, copyright 2022 Elsevier.

The polyphenol-based hydrogel could also be employed to deliver therapeutic miRNA to disease tissues. Wang et al. designed and prepared an injectable gelatin-based inflammatory-responsive hydrogel for the sustained release of curcumin (Cur) and cholesterol-modified miRNA-21 inhibitor (Antagomir-21) to treat intervertebral disc degeneration (IDD) (Figure 8A-E) 91. This study provided a supramolecular host-guest system for miRNA delivery by grafting *β*-cyclodextrin (*β*-CD) onto Gel-phenylboronic acid (BA) (Gel-BA-CD) to form hydrogels with TA. Specifically, *β*-CD-modified Gel-BA bound with TA possessed a large number of natural catechol/phthalate groups to form reversible boronic ester bonds. Cur was encapsulated into the ROS-responsive micelles that were prepared from the amphiphilic polymer mPEG-TK-PLGA (MIC@Cur). Owing to the homogenously porous structures of the hydrogels, Antagomir-21 and MIC@Cur were loaded in Gel-BA-CD, which ensured that the hydrogels have injectable, remolding, and self-healing features. In the context of low pH and high ROS levels, the hydrogel was collapsed, Antagomir-21 was sustainably released due to the reversible host-guest assembly, and MIC@Cur was further collapsed under the condition of overexpressed ROS to rapidly release the anti-inflammatory Cur. *In vitro*, the hydrogel system effectively reduced the inflammatory responses in macrophages and Antagomir-21 down-regulated extracellular matrix (ECM) degradation, recovering the synthesis/catabolism balance of ECM in nucleus pulposus cells (NPCs). Notably, the hydrogel presented a significant inhibitory activity of ROS generation owing to the anti-oxidant effects of Cur and TA, which efficiently promoted the M2 macrophage polarization, thereby relieving the inflammatory response. In the degenerated disc model, Hydrogel&MIC@Antagomir-21 exhibited sustained release of Antagomir-21 and on-demand release of Cur, thus exerting anti-inflammatory activities, reversing the ECM metabolic imbalance and effectively inhibiting the deterioration of IDD, this suggested that this hydrogel system was a potential and effective platform for gene delivery and could also be utilized in other inflammatory tissue repairs.

#### 3.2.3 Polyphenol-assisted dsRNA delivery

Besides, RNAi-based methods are also being explored for controlling insects that carry diseases (such as malaria) and damaging crops. Palli et al. designed a simple, effective, and easy-to-synthesize dsRNA nanosystem (NP_PLL/EGCG/dsRNA_) aiming at controlling pests 92. NP_PLL/EGCG/dsRNA_ was composed of a negatively charged core complexed by dsRNA, EGCG and a PLL-coated shell. Compared with NP_PLL/dsRNA_, a compact spherical nanoparticle with a small size was formed via enhancing dsRNA compressibility, and NP_PLL/EGCG/dsRNA_ showed superior efficiencies of dsRNA delivery owing to the addition of EGCG. It also showed better stability in *Spodoptera frugiperda* (Sf9) cell conditioned medium containing nuclease and improved target gene knockdown efficiency compared to naked dsRNA or PLL/dsRNA. In Sf9 cells, NP_PLL/EGCG/dsRNA_ could effectively protect dsRNA from double-stranded ribonuclease (dsRNase) degradation, increase the tolerance of dsRNA to dsRNase, enhance cellular uptake and endosome escape, thereby resulting in a stronger gene silencing effect.

#### 3.2.4 Polyphenol-assisted mRNA delivery

**Figure 9:**
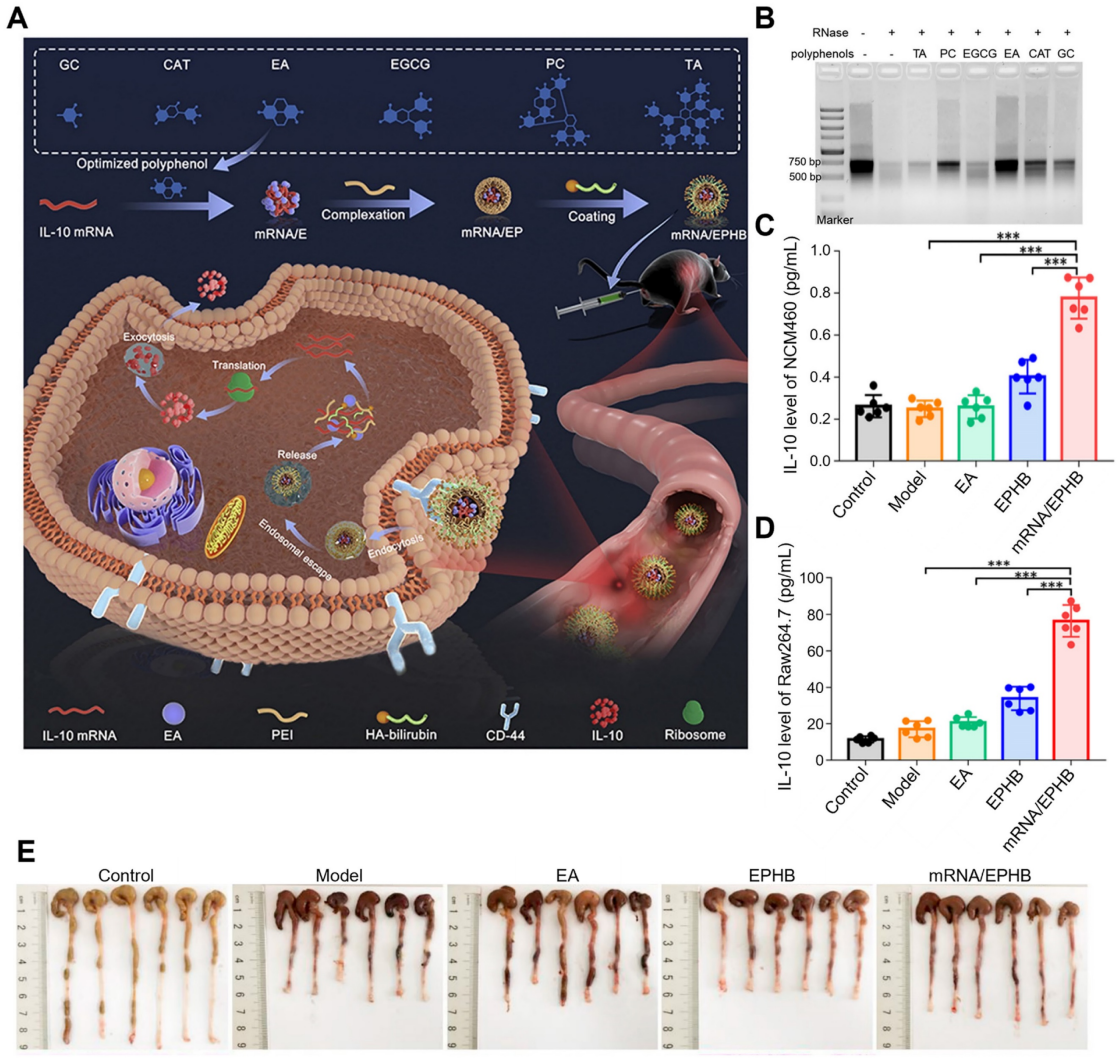
Polyphenol-assisted mRNA delivery. (A) Schematic illustration of the polyphenol-assisted IL-10 mRNA delivery system with self-protective and active-targeted mechanisms. (B) Screening of polyphenols in protecting mRNA degradation. (C) The expression level of IL-10 in NCM460 cells of different groups. (D) The expression level of IL-10 in Raw264.7 cells of different groups. (E) Photos of colon length of different groups after 9-day administration in a chronic UC model. Adapted with permission from 93, copyright 2022 Elsevier.

A Previous study has established an easy, effective, and feasible polyphenol-assisted gene delivery system for UC therapy, in which EA was utilized as a carrier to directly deliver mRNA (Figure 9A-E) 93. This nanosystem (mRNA/EPHB) was constructed layer-by-layer. The negatively charged core was prepared by the binding between IL-10 mRNA and EA (mRNA/E), then the core was complexed with linear polyetherimide (mRNA/EP), and the outermost layer was covered by bilirubin-modified hyaluronic acid (HA-BR). Due to the layer-by-layer structure and the targeting of CD44 of HA, this novel IL-10 mRNA delivery system (mRNA/EPHB) was endowed with active targeting and self-protection, showing a remarkable therapeutic effect in DSS-induced acute UC models. Firstly, mRNA was mixed with 6 frequently used polyphenols (EA, gallic acid (GC), punicalagin (PC), CAT, TA and EGCG) to obtain mRNA/polyphenol complexes. Among phenolics, EA showed the strongest protective effect on mRNA through the assessment of Tyndall effects, ethidium bromide (EB) competitive binding experiment and RNase degradation assay. The hydrodynamic size of mRNA/EPHB was at 229.0 ± 4.8 nm. In NCM460 and Raw264.7 cells, the expression level of IL-10 was significantly enhanced by mRNA/EPHB, which was 8-fold higher than the control group in Raw264.7 cells and 4-fold higher in NCM460 cells. In DSS-induced acute UC model, mRNA/EPHB treatment showed lower DAI score, less weight loss, and longer colon length when compared to the model, EPHB, and 5-aminosalicylic acid (5-ASA) groups. In addition, the successful delivery of IL-10 mRNA through this nanosystem evidently promoted the up-regulation of IL-10 expression and inhibited inflammation in acute UC, showing a restorative and anti-apoptotic effect on the colonic epithelium. Similar results were also confirmed in the chronic UC model. This layer-by-layer core-shell delivery system possesses a great feasibility in clinical transformation which provides a new multifunctional design scheme for gene therapy of IBD.

## 4. Conclusions and future perspectives

As one of the most widely distributed plant bioactive compounds, polyphenols possess excellent biomedical activities, such as anti-tumor, anti-oxidant, anti-inflammatory, and anti-viral effects, and serve as nutraceuticals and adjuvant therapeutic agents in food and pharmaceutical industries. Besides, their unique physicochemical features of dense benzene rings and hydroxyl groups prompt them to be a promising drug carrier, which can interact with different materials, including nucleic acids. In this review, we have summarized the recent progress in natural polyphenol-based gene delivery systems which can be divided into two categories, DNA and RNA delivery systems. Besides, natural polyphenols can also serve as the building blocks to form nanoparticles or hydrogels to assist effective gene transfer, thus possessing high gene silencing efficiency or significantly modulating the expressions of bioactive proteins for disease therapy. Studies have shown that the dense phenolic hydroxyl groups and benzene of polyphenols can combine with nucleic acid drugs through intermolecular hydrogen bonds and hydrophobic interactions, respectively 80,81,90, thereby achieving effective preparation of nucleic acid drug nanoparticles. In the process of cellular uptake, nanoparticles formed by the complexation of nucleic acids and polyphenols have the advantage of its size, which is more conducive to the passive targeted uptake of nanodrugs by the cells. In addition, nanostructures produced by the complexation of polyphenols with nucleic acid drugs can also be actively targeted through the coating of functional materials, such as HA with CD44 active targeting 86. Generally, loose nucleic acid nanostructures can be easily hydrolyzed by extracellular rich nucleic acid degrading enzymes. For the stability of nucleic acid drugs, the usual strategy is to use polymers with high charge density and molecular weight, such as PEI, to encapsulate nucleic acid drugs. Although the binding affinity and transfection efficiency of nucleic acid drugs have been improved in these efforts, the efficiency-toxicity correlation of these polymers is unsatisfactory. Currently, there is no direct evidence showing that the involvement of polyphenols in nucleic acid drug delivery systems contributes to intracellular escape. The main role of polyphenols in nucleic acid delivery systems is to improve the affinity between nucleic acid drugs and delivery carriers, reduce the risk of hydrolysis of nucleic acid drugs *in vitro* and the safety risks of high potential density and high molecular weight carriers. In this review, the preparation of polyphenol-based gene delivery system was described briefly. Besides, the *in vitro* gene delivery efficiency and *in vivo* therapeutic effects for various diseases were highlighted, this provided an easily accessible guide for the research and application of natural polyphenol-based gene delivery.

Despite considerable potential outcomes that have been achieved in polyphenol-assisted gene delivery systems, there are some challenges that need to be solved: 1) Nucleic acids are susceptible to enzyme degradation in the physiological environment due to their circulatory instability. Besides, it is a double-edged sword that polyphenols could interact with biomacromolecules (nucleic acids, proteins, and polysaccharides). Polyphenols can bind with genes to form polyphenols-based gene delivery vectors, but they can also interact with the extracellular matrixes of organs and tissues, thus leading to decreased circulatory stability. 2) The difficulty in storage. Natural polyphenols are unstable and can be easily oxidized into oligomers and polymers in the air 60. Therefore, polyphenol-gene complexes should be freshly used or not be stored for a long time after fabrication. 3) The difficulty in achieving clinical application. Although natural polyphenols are demonstrated as a safe, easy-obtained, and cheap vector for gene therapy, their low water solubility, instability, and poor targeting are the most obvious disadvantages of polyphenols as a building block for gene delivery systems. At present, only a few polyphenols are found to be used as gene carriers, and the carrier type is relatively undiversified (nanoparticles and hydrogels), which heavily hinder their clinical applications 59. In addition, polyphenol-based gene delivery systems are complex and usually consisted of several components. Therefore, the mechanisms of cellular uptake, adsorption, metabolism, and excretion of polyphenol-based gene delivery systems *in vivo* are not fully understood, which may affect the safety of clinical applications. In conclusion, polyphenols-based gene delivery is a promising strategy for effective delivery of nucleic acid drugs, but there is still a long way to be applied in the clinic.

## Abbreviations

ADA: adenosine deaminase
Antagomir-21: cholesterol-modified miRNA-21 inhibitor
APTES: (3-aminopropyl)-triethoxysilane
5-ASA: 5-aminosalicylic acid
ASO: antisense oligonucleotide
BA: boronic acid
*β*-CD: *β*-cyclodextrin
Bcl-xL: B-cell lymphoma-extra large
b-MPN NPs: bioactive metal-polyphonic nanoparticles
CAT: catechin
CD44: cluster of differentiation 44
cIAP1: cellular inhibitor of apoptosis protein 1
CTGF: connective tissue growth factor
Cur: curcumin
DAI: disease activity index
DBC: DNA blocking copolymer
dsiRNAs: dicer substrate RNAs
dsRNA: double strand RNA
dsRNase: double-stranded ribonuclease
DSS: dextran sulfate sodium
EA: ellagic acid
EB: ethidium bromide
ECG: epicatechin gallate
ECM: extracellular matrix
EGC: epigallocatechin
EGCG: epigallocatechin gallate
EIF5A2: eukaryotic translation initiation factor 5A2
GA: gallic acid
GAPDH: glyceraldehyde 3-phosphate dehydrogenase
GNPs: green nanoparticles
GSH: glutathione
HA: hyaluronic acid
HAase: hyaluronidase
HFV: human foamy virus
IDD: intervertebral disc degeneration
IECs: intestinal epithelial cells
LPF: Lipofectamine 2000
miRNA: microRNA
MMP-9: matrix metallopeptidase 9 protein
MNC: magnetic nanocrystal clusters
MPNs: metal-polyphenol networks
mRNA: messenger RNA
NaCl: sodium chloride
NPCs: nucleus pulposus cells
PARP: poly ADP-ribose polymerase
PC: punicalagin
pDNA: plasmid DNA
PEI: polyethylenimine
p-ERK: phospho-extracellular regulated protein kinases
p-FAK: phosphor-focal adhesion kinase
PHD2: prolyl hydroxylase 2
PLL: ε-polylysine
Ps-ASO: phosphorothioate backbone ASO
P13K/Akt: phosphoinositide 3-kinase/ protein kinase B
RNAi: RNA interference
ROMP: ring opening metathesis polymerization
SCID: severe combined immunodeficiency
Sf9: *Spodoptera frugiperda*
shRNAs: short hairpin RNAs
siLuc: siRNA targeting luciferase
siRNA: short interfering RNAs
siRNA-CTSK: siRNA cathepsin K
TA: tannic acid
TE: TransExcellent-siRNA
UC: ulcerative colitis
Y-DNA: Y-shape DNA
